# Medical economic vulnerability: a next step in expanding the farm resilience scholarship

**DOI:** 10.1007/s10460-022-10307-4

**Published:** 2022-02-16

**Authors:** Florence A. Becot, Shoshanah M. Inwood

**Affiliations:** 1 National Farm Medicine Center, Marshfield Clinic Research Institute, 1000 N Oak Ave, Marshfield, WI 54449 USA; 2 School of Environment and Natural Resources, The Ohio State University, 132 Williams Hall, 1680 Madison Avenue, Wooster, OH 44691 USA

**Keywords:** Farm resilience, Household level difficulties, Health insurance, Medical economic vulnerability, Objective vs. subjective measures, Agri-family system

## Abstract

In recent years, the long-standing questions of why, how, and which farm families continue farming in the face of ongoing changes have increasingly been studied through the resilience lens. While this body of work is providing updated and novel insights, two limitations, a focus on macro-level challenges faced by the farm operation and a mismatch between the scale of challenges and resilience measures, likely limit our understanding of the factors at play. We use the example of medical economic vulnerability, a micro-level challenge traditionally confined to the household sphere of the agri-family system, as a way to call attention to these limitations. Focusing on United States (U.S.) farm households, we assess: (1) To what extent are they experiencing medical economic vulnerability when using objective and subjective outcome measures? (2) Which demographic and farm characteristics are associated with experiencing medical economic vulnerability? (3) What is the association between institutional arrangements and medical economic vulnerability? Our analysis of over 900 surveys coupled with a conceptual framework merging complementary insights from three bodies of literature revealed seemingly large differences in the prevalence of medical economic vulnerability across the objective and subjective measures with the subjective measure indicating a general sentiment of medical economic vulnerability in a majority of respondents. Conversely, limited variations were noted in who experiences medical vulnerability on the basis of demographic and farm characteristics, with stronger associations being connected to the households’ health insurance arrangements. We conclude with three implications of our findings for the farm resilience literature.

## Introduction

In life and agriculture, the one certainty is change. The inherent instability of the agricultural sector associated with biophysical processes and the influence of social, political, and economic forces on the sector have long led rural social scientists to study why, how, and which farm families stay on the land in the face of unrelenting change. Depending on geographical context and time-period, these questions have historically been examined through several strands of literature, most notably the “agrarian question” and “farm persistence” (e.g. Akram-Lodhi and Kay 2010a, b; Buttel 2001; Reinhardt and Barlett 1989). In recent years, these questions have increasingly been studied through the “farm resilience” lens (e.g. Darnhofer et al. 2016; Kangogo et al. 2020; Sinclair et al. 2014). Setting aside some of the theoretical scrutiny on the definition and usefulness of the resilience concept (for these debates see for example Calo 2020; Darnhofer et al. 2010; Hall and Lamont 2013), the farm resilience literature is broadly concerned with farm systems’ adaptation and transformation to maintain their function in the long-term in response to vulnerabilities in the short- and medium-term. In turn, vulnerabilities, also commonly referred to as challenges, are the specific perturbations that negatively impact the functioning of farm systems (Darnhofer et al. 2016; Meuwissen et al. 2019; Urruty et al. 2016).

Several drivers are likely spurring the growth of the farm resilience literature. The interdisciplinary grounding of the concept considers the dynamic interdependences of natural and social systems (Darnhofer et al. 2016) and reflects the growing trend in transdisciplinary team science. This approach lies in contrast to the social systems focus of the farm family literature in the late twentieth century. The growth of the farm resilience literature may also be explained by the omnipresence of the concept of “resilience” itself in governmental and non-governmental organizations discourses (e.g. USDA 2021; World Bank n.d.). However, in our review of the farm resilience literature focused on Western industrialized countries, we identified two limitations that may hinder a holistic understanding of: (a) the range and frequency of challenges that farm families face, and (b) the range of factors that shape farm family resilience. As this body of work continues to evolve, it is helpful to reflect on how these knowledge gaps likely constrict our understanding of the types of interventions that might best support the people in the farm sector and their ability to weather crises.

The first limitation is connected to a doubly narrow prevailing conceptualization of what constitutes stresses and shocks (hereafter described as challenges). On one hand, farm resilience scholars tend to focus on macro-level challenges which impact many farms at once within a geographic area may it be at the sub-national, national, or international scale. These include major weather events (Javadinejad et al. 2020), political and economic structural shifts such as agricultural deregulation (Forney and Stock 2014), and public health crises such as Coronavirus Disease 2019 (COVID-19; Darnhofer 2020). Micro-level challenges that may only impact a few farms at once within a geographical area, such as a barn fire or a wild animal attack of livestock, have received much less attention. Yet, similar to macro-level challenges, these micro-level challenges have short-term impacts on the farm operation due to the loss and redirection of resources (i.e. money and labor) with potential long-term consequences on farm resilience. One the other hand, farm resilience scholars largely focus on challenges that impact the farm operation. Seldom do they consider those faced by farm households such as a major illness, divorce, or loss of off-farm employment (Komarek et al. 2020; Popp and Nowack 2020). The lack of attention to these household-level challenges and how they may affect farm resilience is surprising given the farm resilience studies that have documented the key buffering functions played by farm households within the multi-scalar farm system, as the deep interconnections and exchange of resources between the farm household and the operation help to absorb shocks to the enterprise (Doeksen and Symes 2015; Meuwissen et al. 2019).

The second limitation is connected to the contradiction and mismatch in the scale at which challenges and resilience indicators are measured. While much of the farm resilience research focuses on macro-level challenges, studies tend to emphasize the micro-level variables associated with vulnerability and resilience, such as farmers socio-demographic characteristics, farm operation characteristics, farmers’ actions and pre-disposition, and their adaptation strategies (Darnhofer et al. 2016; Daugstad 2019; Diserens et al. 2018; Greenhill et al. 2009; Kangogo et al. 2020). As scholars critiquing the resilience lens have argued (speaking about the application to agriculture and other topics), the de facto interpretation of this micro-level focus is to interpret resilience through an individual’s deficits (Calo 2020; Cote and Nightingale 2012; Hall and Lamont 2013; Joseph 2013). Often missing are factors outside of farmer’s control that affect their decision-making and shape farm resilience. Meso and macro-level variables such as community infrastructures, rural labor markets, or government programs have received limited attention even though these institutional arrangements play an important role in amplifying or mitigating the effects of challenges that farm families face (Greenhill et al. 2009; Popp and Nowack 2020; Thorsøe et al. 2020). Without considering how meso and macro-level factors shape farm resilience, we limit our ability to assess the interplay and interactions between micro, meso, and macro level factors, and we constrict our ability to understand how power differentials across these different scales affect short- and long-term farm resilience.

In this article, we use the example of medical economic vulnerability among United States (U.S.) farm families to speak to the two limitations of the farm resilience literature. Our article also connects to recent calls to expand the range of challenges studied (Komarek et al. 2020; Meuwissen et al. 2019; Popp and Nowack 2020) and to expand our understanding of the factors that shape these challenges including through the assessment of system’s features that can enable or constrain a farmers’ ability to adapt (Darnhofer 2021). Medical economic vulnerability (i.e., experiencing economic and social difficulties due to health expenses) is illustrative of a micro-level challenge that is generally confined to the household and one for which institutional arrangements in the form of health insurance likely play an important role in softening the blow of a medical crisis. Our three research questions are: (1) To what extent are U.S. farm households experiencing medical economic vulnerability when using objective and subjective measures of vulnerability? (2) Which demographic and farm characteristics are associated with experiencing medical economic vulnerability? (3) What is the association between institutional arrangements, namely health insurance, and medical economic vulnerability? We draw on a primary data set of over 900 surveyed farm households from 10 states along with secondary data on health insurance and labor market environments. Our conceptual framework is based on three distinct bodies of literature which brought together speak to the range of factors within the multi-scalar agri-family system that shape medical economic vulnerability. Furthermore, our comparison of objective and subjective outcome measures provide a multi-dimensional perspective into the frequency and factors that shape farm families’ vulnerability.

Our presentation of the empirical case is preceded by a literature review on health challenges in agriculture and interactions with the farm operation in addition to providing a description of our medical economic vulnerability conceptual framework.

## Literature review: health challenges and medical economic vulnerability in agriculture

Health challenges are common in agriculture as it ranks as one of the most dangerous occupations globally due to the risky nature of the worksite (Donham and Thelin 2016; International Labor Organization 2014; Shortall et al. 2019). The physical nature of the work also takes a toll on the body with the proportion of physical limitations increasing with age (Peters et al. 2008; Reed 2008). Besides impacting farmers’ quality of life, these health aliments, whether they are temporary or permanent, negatively impact the farm business. Health difficulties may limit the tasks farmers can do, which can lead to a decrease in productivity and early farm exit (Chang et al. 2011; Inwood et al. 2018). Depending on the availability and strength of the social safety net, health-related expenses can also represent a source of economic pressure for the farm as health-related expenses can divert resources away from the farm operation while loss of work time and cost of replacement labor can impact farm income (Dulitz and Schrader 2013; Inwood 2015; Lottero et al. 2007).

Research from low and medium income countries highlights the importance of considering health challenges along with the macro-level structural challenges that impact the farm operation (Alam and Mahal 2014; Bonfrer and Gustafsson-Wright 2017). This body of work has found that accidents and illnesses can be more frequent than farm-level challenges such as crop failures and major storms and they can affect anyone no matter the scale of operation, commodities produced, climatic conditions, or social policy environments. Furthermore, health insurance plays a crucial role in supporting the farm household and operation by removing, or at least softening, the blow of health related-expenses.

Overall, previous research on medical economic vulnerability among farmers in Western industrialized countries comes from the U.S. and largely pre-dates the 2010 Patient Protection and Affordable Care Act (ACA^1^). This points to a gap in our understanding of the ways in which health challenges may impact farm resilience. U.S. studies highlight the problem of underinsurance among farmers (i.e., a situation when health insurance coverage does not offer adequate financial protection). A 2007 survey of 2017 Midwest farmers found that while over 90% of farmers had health insurance coverage, 18% had a medical debt (Pryor et al. 2009). Meanwhile, a 2012 survey of 205 South Dakota farmers found that 91% had health insurance yet 62% reported making sacrifices due to health expenses (Dulitz and Schrader 2013). Though these challenges might not be as acute in other Western industrialized countries as they are in the U.S. due to stronger social safety nets, there is international evidence that some farmers are experiencing difficulties accessing and paying for health care. For example, French farmers reported difficulties associated with low reimbursement rates for some health services while Swiss farmers faced challenges associated with high health insurance costs and out-of-pocket expenses (Chappuis et al. 2015; Droz et al. 2014; Roche 2016).

The limited consideration of medical economic vulnerability among farm households and the ways in which health challenges can affect the farm operation and ultimately farm resilience may be partially attributed to difficulties accessing data on farmers’ health and health insurance data as previously noted (Chang et al. 2011; Droz et al. 2014). The U.S. Department of Agriculture (USDA) collects data on source of health insurance coverage and health care expenditure. Yet the lack of data on health status and lived experiences along with limited demographic data have largely limited research to interactions between health insurance coverage with labor allocations and off-farm employment (e.g., Ahearn et al. 2013; Mishra et al. 2018). In this study, we leverage our access to a primary dataset of U.S. farm families to which we add secondary data on health insurance and labor markets to assess the prevalence and factors associated with medical economic vulnerability.^2^

## Conceptual framework

**Fig. 1:**
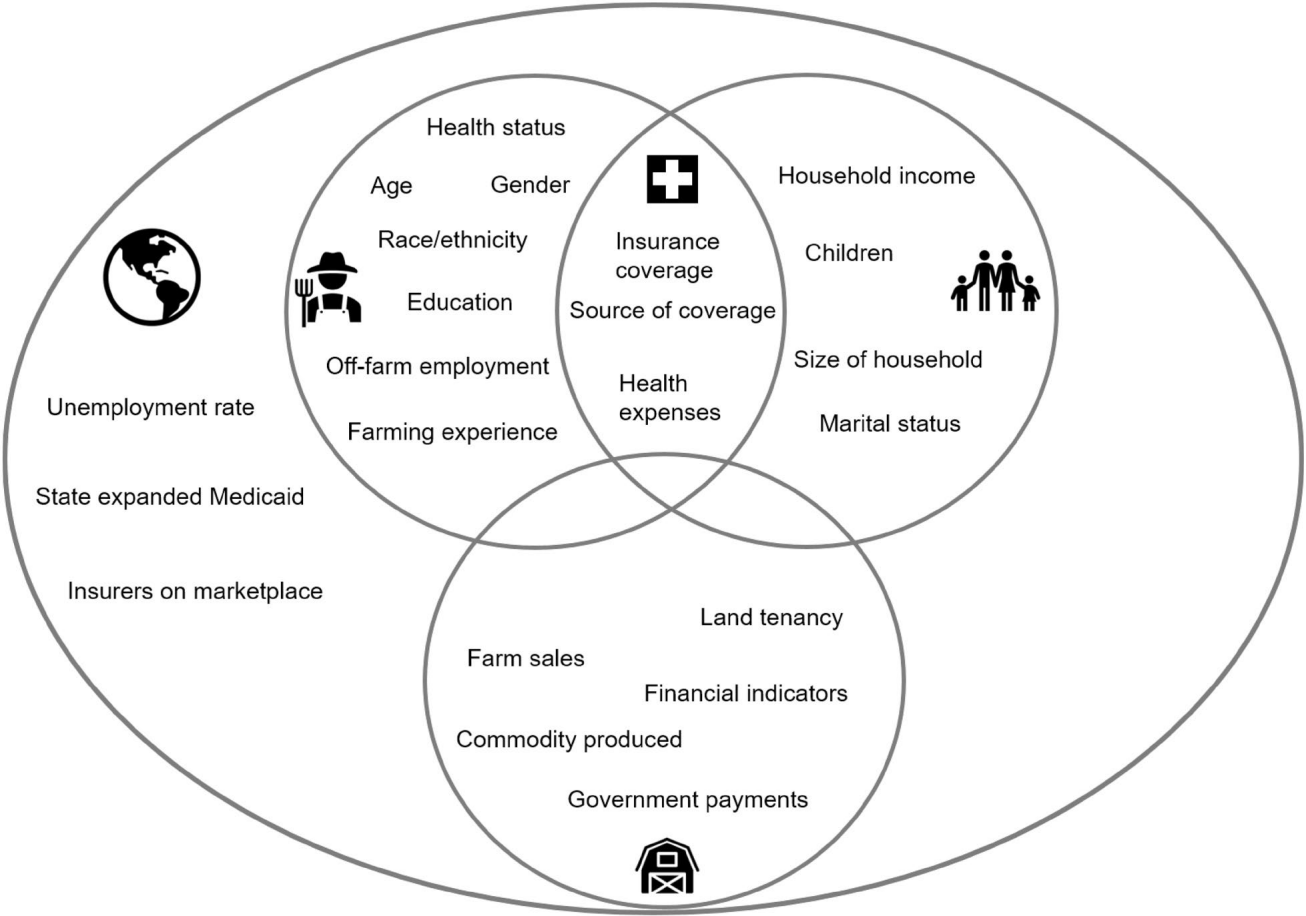
Conceptual framework of farm families’ medical economic vulnerability. *Note* conceptual framework variables are from the merging of three bodies of literature: (1) medical economic vulnerability in the U.S., (2) farm economic stress and bankruptcy literature (largely based on the U.S. literature), and (3) farm family literature, including farm resilience

Given the limited number of studies examining farm families’ medical economic vulnerability, we developed our conceptual framework by merging three distinct, but complementary, bodies of literature: (1) medical economic vulnerability in the U.S., (2) farm economic stress and bankruptcy literature (largely based on the U.S. literature), and (3) farm family literature, including farm resilience. First, we identified and summarized the independent variables associated with medical and farm economic vulnerability. Then, we drew on the farm family literature, in particular its description of the complex-agri family system, as a backdrop to organize the variables from the medical and farm economic vulnerability bodies of literature (Fig. 1).

By showing the individual (), farm household (), and farm operation () as overlapping spheres, our conceptual framework underscores the interactions between these micro-level spheres within the agro-family system (Bennett and Kohl 1982; Doeksen and Symes 2015; Komarek et al. 2020; Meuwissen et al. 2019; Popp and Nowack 2020; Reinhardt and Barlett 1989). By showing the micro-level spheres embedded within the larger environments connected to meso- and macro-level spheres (), our framework underscores the ways in which larger environments intersect with farm families’ choices and access to resources (Bennett and Kohl 1982; Meuwissen et al. 2019; Popp and Nowack 2020; Smithers and Johnson 2004; Thorsøe et al. 2020). We inserted the variables associated with health insurance arrangements () at the intersection of the individual and farm household spheres since eligibility criteria and cost of coverage are heavily shaped by the characteristics of these two spheres. As with most graphical representations, our conceptual framework is simplified to enhance readability and streamline operationalization. This simplification includes the merging of meso- and macro-level spheres into one and placing certain factors into one sphere even though they could have been placed in multiple spheres.

While this conceptual framework was developed to study farm families’ medical economic vulnerability, our framework also speaks to the limitations of the farm resilience literature highlighted in our introduction. By speaking to micro-, meso-, and macro-level variables within the agri-family system, our framework provides an example of how multi-scalar factors can be better integrated into farm resilience research. The consideration of variables across scale provides an opportunity to further our understanding of the interplay between individual variables and systems’ features. We now summarize the factors associated with medical and farm economic vulnerability related to the components of our conceptual framework.

### Health insurance arrangements

The U.S. medical economic vulnerability literature not only points to the importance of having health insurance but also to the type of coverage. Medical economic vulnerability is more likely to occur when households do not have health insurance, have experienced a gap in coverage, and have high out-of-pocket and deductible expenses (Banegas et al. 2016; Baughman et al. 2015; Hamel et al. 2016; Himmelstein et al. 2009; Pryor et al. 2008, 2009). The source of coverage associated with vulnerability is not consistent across studies. Hamel et al. (2016) and Pryor et al. (2008) found a greater probability of medical economic vulnerability for those covered through a private plan with high deductibles or purchased on the individual marketplace, while Banegas et al. (2016) found a greater probability among those who were insured through a public plan.

## Individual and household characteristics

The medical economic vulnerability and farm economic stress and bankruptcy bodies of literature indicate a number of individual and household level characteristics associated with economic vulnerability. Individuals more likely to be economically vulnerable have poorer health status, have a pre-existing or chronic health condition, are female-headed households and/or operated farms, are African American, have children, have larger households, have lower educational attainment, have lower household income, and are beginning farmers (< 10 years of experience) (Banegas et al. 2016; Baughman et al. 2015; D'Antoni et al. 2009; Katchova and Dinterman 2018; Nadolnyak et al. 2019; Pryor et al. 2009; Wiltshire et al. 2016).

Findings on the association between income source and age are contradictory. In a study of medical economic vulnerability, Pryor et al. (2008) found that full-time farming households (i.e. those without off-farm employment) were more vulnerable than those farming part-time. Looking at the financial stress of beginning farmers, D'Antoni et al. (2009) found that financial hardship decreased as the share of income from farming increased. While some studies found that younger households are more likely to be vulnerable, others did not find age-related associations (Baughman et al. 2015; D'Antoni et al. 2009; Hamel et al. 2016; Himmelstein et al. 2009; Katchova and Dinterman 2018; Pryor et al. 2008, 2009). This discrepancy in findings around age reinforces the need to consider household composition and life course variations while also considering health insurance arrangements since in the U.S., access to some public plans and the ability to be covered by a parents’ insurance have an age criterion. While not mentioned in the farm economic stress and bankruptcy studies, the broader farm family literature has also found that farming background (i.e., whether someone grew up on a farm) shapes access to financial resources and social networks with multi-generational farmers having greater access to resources than first-generation farmers (Carolan 2018; Clark et al. 2010; Inwood et al. 2013).

## Farm operation characteristics

Farm operations that are more likely to be economically vulnerable include small- and large-scale operations as their sales are either low or their financial leverage is high (Bryant and Maisashvili 2017; D'Antoni et al. 2009; Franks 1998; Katchova and Dinterman 2018). However, there are no clear patterns in the associations between economic vulnerability for farm income, commodities produced, land tenancy, and government payments (D'Antoni et al. 2009; Franks 1998; Katchova and Dinterman 2018; Nadolnyak et al. 2019; Shepard and Collins 1982). This lack of pattern might be due to differences across studies reflecting their variations in geographical areas, outcome measures, or use of aggregate vs. farm operation level data.

## Macro-level environments

The last set of variables is connected to macro-level environments. According to the farm economic vulnerability literature, greater vulnerability is associated with higher unemployment rates at the county level due to the importance of off-farm employment for household income (Dinterman et al. 2018; Nadolnyak et al. 2019). To our knowledge, medical economic vulnerability scholars have not considered the role played by the county- and state-level health insurance environments. However, research assessing the impact of the ACA on health insurance coverage and cost has found increased coverage in states that have expanded Medicaid and relationships between number of insurance plans offered on the marketplace and cost of premiums (Antonisse et al. 2019; Burke et al. 2014; Frank and McGuire 2017; Mazurenko et al. 2018).

## Data and methods

### Research design and data collection

We use a mix of primary and secondary data to answer our research questions. Primary data on health insurance arrangements, individual and household characteristics and farm operation characteristics are from a national survey to understand how health insurance impacts farms and ranches. The instrument was designed using several bodies of literature (health insurance and health care access with a focus on rural areas and the farm population, health literacy, and farm business development) and insights from key informant interviews with University Extension personnel, tax preparers, farm organizations, and state department of agriculture staff. The secondary data examining macro-level county and state level health insurance and labor markets are from publicly available data sets (Henry Kaiser Family Foundation 2019; Robert Wood Johnson Foundation n.d.; USBLS 2016).

We used the tailored design method for mail and online surveys to deploy the survey (Dillman et al. 2014). The surveyed population was farm households in 10 states (California, Kentucky, Massachusetts, Michigan, Mississippi, Nebraska, Pennsylvania, Utah, Vermont, and Washington State) which were selected using two criteria. First, we selected two states in each of the four USDA regions (Northeast, North Central, South, and West) to account for regional and production variations. Then in each region, we selected a state that had expanded Medicaid and a state that had not (except in the Northeast region) to account for health insurance policy variations. We sent introductory letters, multiple mailing and emailing of the survey instruments, and reminder letters February–April 2017 to a purchased list of 10,165 randomly selected farm households. The letter asked that the household member with the most knowledge about health insurance fill out the survey. Paper surveys were entered manually then merged with the online survey responses. We conducted quality control during the data entry and merging processes including checking for accurate data entry and duplicate responses. We received 1292 completed surveys. After removing bad addresses and blanked surveys, the response rate was 13%. Despite the low response rate, our sample of 1292 respondents is above the 384 sample-size threshold for 95–5 confidence level-margin of error since there are 536,000 primary operators living in the 10 study states (U.S. Department of Agriculture 2017). We removed responses from 113 hobby farms (sales under $10,000) from our dataset to focus on farmers that are most likely to be commercially-oriented. For further discussion on the research design, see Inwood et al. (2018). The research protocol was determined to be exempt from review by the University of Vermont Institutional Review Board.

### Measures and recoding

The variables used for analysis along with the descriptive statistics are listed in Table 1, and the independent variables are organized under the headers of our conceptual framework (Fig. 1).

**Table 1:** Study variables and descriptive statistics (n = 1179)

	Percent	Mean (standard error)
Outcome variables: measures of medical economic vulnerability		
Medical debt over $1000^a^ (objective measure)	20.3	
Confidence can pay for major health expenses without going into debt^b^ (subjective measure)		
Not at all or slightly confident	55.5	
Neutral	12.9	
Moderately or very confident	31.7	
Independent variables: health insurance arrangements ()		
Had health insurance for all household members all year	92.5	
All household has same plan	73.1	
Source of health insurance		
Off-farm employment	34.3	
Direct purchase of private plan	33.8	
Age-based public health insurance	21.3	
Means-based public health insurance	9.0	
Farm Bureau or Farmers' Union	5.2	
Monthly insurance premium in 2016		$750.80 ($833.40)
Health insurance deductible		
None	9.2	
$1 to $1999	31.0	
$2000 to $5000	32.9	
More than $5000	27.0	
Out-of-pocket expenses		
Up to $999	22.1	
$1000 to $2999	26.1	
$3000 to $4999	21.4	
$5000 and over	30.4	
Health savings account	22.8	
Flexible spending account	8.3	
Independent variables: farm individual () and household ()		
Pre-existing or chronic condition	59.5	
Age of respondent		57.9 years (11.3 years)
Children under 18	23.3	
White, non-Hispanic/Latino	95.7	
Female	38.7	
Education		
High school or less	37.3	
Some college	20.8	
Bachelor's degree and higher	42.9	
Off-farm job	47.2	
Beginning farmer	7.7	
Independent variables: farm operation ()		
Farm sales		
Small	39.7	
Medium	21.7	
Large	38.6	
Multi-generational farm	77.0	
Commodity produced		
Grain	53.4	
Livestock	37.9	
Dairy	20.0	
Fruits and vegetables	13.8	
Independent variables: health insurance and labor market () environments		
State expanded Medicaid	56.8	
Number insurers on marketplace		6.8 (3.3)
Unemployment rate		4.6 (2.0)

^a^Having a medical debt over $1000 is used as the outcome variable in the objective measure of medical economic vulnerability model (model 1) and as an independent variable in the subjective measure of medical economic vulnerability model (model 2)

^b^Variable is collapsed into a dummy variable for the bivariate and multivariate analysis (“Not confident could pay for major health expenses without debt” vs. “neutral or confident”)

#### Outcome variables

We used objective and subjective measures to assess medical economic vulnerability. Our rationale for using two types of measures is justified by the bodies of literature that underpin our conceptual model. The farm resilience and farm economic stress bodies of literature have been critiqued for their overreliance on objective measures which prevent the development of a holistic understanding that incorporates farmers’ perceptions, goals, and lived realities (see Darnhofer et al. 2016; Kuhmonen 2020; Meuwissen et al. 2019; Perrin et al. 2020 for critiques of the farm resilience literature and Gillespie and Johnson 2010; Rissing 2019 for critiques of the agricultural economics literature). The medical economic vulnerability literature reinforces the importance of incorporating subjective measures because the perception of economic vulnerability has been associated with poor mental and physical health outcomes (Richardson et al. 2013; Sweet et al. 2013).

Our objective measure is “having a medical debt over $1,000”; the $1000 debt threshold was selected based on previous medical bankruptcy research (Himmelstein et al. 2009). Collected as a dummy variable, this response did not require recoding. Our corresponding subjective measure is based on the question “Given your current financial and health insurance situation, how confident are you that you could pay the medical costs, without going into debt, if you had a major illness or injury such as heart attack, cancer, or loss of limb?” This response was initially measured using a five-point Likert scale and collapsed to three categories to ensure we had at least five observations for analysis in the dependent/independent variable cross-tabulations. Since our ordinal logistic model with survey responses on the three-point scale failed the proportionality odds assumption test, we ultimately chose to recode this variable as a dummy variable (not confident vs. neutral or confident) for two reasons. First, while a multinomial logistic model is generally recommended when the proportionality odds assumption is not met in the ordinal logistic model, the interpretation of multinomial logistic models results is cumbersome. Second, by having dummy objective and subjective measures, we can more easily compare the results of our two models.

Similar to the limitations associated with the use of unidimensional variables in farm resilience and farm economic stress assessments, the use of two dummy variables to assess medical economic vulnerability is a limitation of our study design. In an attempt to address this limitation, we conducted an exploratory factor analysis (EFA) to create a composite measure for the subjective response using three survey questions.^3^ EFA results reached satisfactory thresholds but missing observations led to an important drop in the sample size in the multivariate analysis. Therefore, we elected to conduct our assessment using one subjective measure. Future work aimed at furthering the development of multidimensional measures of resilience and vulnerability is needed.

#### Independent variables

The independent variables speak to our conceptual framework. Starting with health insurance arrangements, measures include insurance coverage in the previous year, source of coverage, costs (i.e., premiums, deductible, and out-of-pocket expenses), and health savings tools [i.e., health savings account (HAS) and flexible spending accounts (FSA)].^4^ We collapsed the insurance coverage variable from three to two categories (all household members had insurance all the time in the previous year vs. household members had no coverage or partial coverage) to satisfy the five observations per cell requirement of our selected analysis. The health insurance deductible and out-of-pocket expenses variables were collapsed from seven to four categories. Because having a medical debt is likely associated with the degree of confidence in the ability to pay for major medical expenses without going into debt, we added the medical debt variable as an independent variable in the subjective measure model.

The farm individual variables include age, gender, race/ethnicity, and educational attainment while household level variables include health status (i.e. household member has a pre-existing or chronic health condition), children under 18, off-farm employment, and beginning farmer status. Since some of the demographic variables were collected only for the survey respondent, we are unsure of the racial/ethnic makeup of the entire farming household. We collapsed the race and ethnicity variables from seven to two categories (i.e., White, non-Hispanic/Latino vs. Farmer of Color) and educational attainment from five to three categories.

The farm operation variables include farming background (i.e., parent or other relatives ran the farm before respondent), commodity produced, and farm sales. We collapsed farm sales from eight to three categories using the USDA farm size categories based on farm sales (small, medium, and large).^5^ While previous farm economic stress studies have collected data on land tenancy, farm financial indicators, and government payments, we did not collect these measures.

Last, publicly available data connected to health insurance and labor market environments for the study states were merged to the survey data using state and ZIP code as the merging variables. This included Medicaid expansion status (Henry Kaiser Family Foundation 2019), number of health insurance plans on the state and county market places (Robert Wood Johnson Foundation n.d.), and county-level unemployment rate (USBLS 2016).

A limitation to our study is that the survey instrument did not include independent variables that are associated with vulnerability in the medical economic vulnerability and farm economic stress literatures (i.e. household income, land tenancy, farm financial indicators, and government payments). To address the significance of household income as a factor, we assessed potential specification errors by running our preliminary multivariate analysis with and without a household income estimate from the USDA.^6^ Based on the model fit tests, coefficients, and standard errors, there were no noticeable differences between the models with and without the household income estimate. Furthermore, the linktest, a STATA function to test for additional predictors that are not statistically significant other than by chance, indicated no misspecification error in our models.

Another study limitation is our greater use of household’s health insurance arrangements variables over health insurance environment variables. The U.S. health insurance system is a complex patchwork of options from the public, private, and/or non-profit sectors with an emphasis on consumers’ choice (Rice et al. 2013). While some could argue that micro-level variables prevents us from assessing the role of institutional arrangements, scholars have pointed to the illusion of choice as health insurance choices are driven by a complex set of drivers largely outside of consumer’s choices (Mulligan et al. 2019). As such, the specifics of households’ health insurance are connected to institutional arrangements. For example, for households with coverage through off-farm employment, the employer chooses the coverage they offer which is determined by what private insurers in their area offer.

## Analytical strategy

We first conducted bivariate analysis to assess the prevalence of medical economic vulnerability using the objective and subjective measures as outcomes variables. We then conducted logistic regression analysis to assess the factors associated with medical economic vulnerability (model 1: objective measure; model 2: subjective measure; model 3: subjective measure with the addition medical debt as an independent variable). We clustered the standard error at the state-level in the multivariate models to account for the multilevel conceptual framework.^7^ We used a nested modelling approach in the subjective outcome model to test the importance of the debt variables in the perception of medical economic vulnerability. The likelihood ratio test comparing models 2 and 3 indicates that the model fit is improved when the medical debt variable is added in model 3 (p = 0.042). Last, because health care needs vary across the life course and eligibility criteria for universal public insurance coverage is age-based (i.e., age 65), we first added age as a quadratic term in the models to assess curvilinearity in the relationship between medical economic vulnerability and age. The significance levels for the age square term were above 0.05 in the three models indicating that the relationship between the dependent and independent variables is linear. Therefore, we did not include age as a quadratic term in the final models. Model diagnostics indicate that the three models have acceptable fit for the data based on the Hosmer–Lemshow test, there are no specification errors based on the linktest, no multicollinearity [mean variance inflation factor (VIF) was ~ 1.52 across the three models and maximum VIF value was 2.77], and there are no major influential observations based on Pearsons and deviance residuals.

To limit potentially biased estimators from missing observations, we conducted the bivariate and multivariate analysis on imputed datasets (He 2010; van Ginkel et al. 2020). Between 47 and 52% of the observations did not have any missing values in the logistic models. The highest proportion of missing values was for the ‘health insurance premium’ variable with 34% of observations missing while 6% of observations were missing the subjective outcome measure. We used the multiple imputation by chained equation (MICE) approach, which accounts for categorical and dummy variables, with 35 iterations (Royston and White 2011; UCLA n.d.; White et al. 2011). All model variables were included in the imputation model but we did not impute the outcome variables values (Allison 2012; UCLA n.d.). We polled the imputed datasets for analysis using the Rubin’s combination rule (Carlin et al. 2008). Our imputed datasets included 1009 and 993 observations, respectively, for the objective and subjective measures models compared to 617 and 616, respectively, for the unimputed analytical datasets. We conducted the imputation and data analysis in STATA IC (version 16) using the “mi” suite of commands.

The model F-tests on the imputed data indicate that in the three models, at least one of the independent variables is different from 0 meaning that the models are better fit than those with no predictors (model 1: F = 48.81, p = 0.000, adjusted R^2^ = 7.7%; model 2: F = 97.80, p = 0.000, adjusted R^2^ = 11.3%; model 3: F = 90.43, p = 0.000, adjusted R^2^ = 11.7%). We assessed statistically significant differences across variables using χ^2^, ANOVA, and t-tests with the threshold level of significance set at p ≤ 0.05.

## Results

### Characteristics of the sample

The characteristics of the sample are available in Table 1. Starting with the health insurance arrangement variables, 92.5% of respondents reported that all household members were covered by health insurance all year, and 73.1% reported having one insurance plan for their household. The most frequent source of coverage was through off-farm employment (34.3%) followed by a privately purchased plan (33.8%), and age-based public health insurance (i.e., Medicare) (21.3%). Respondents spent on average $750.8 in monthly health insurance premiums in 2016 (standard deviation $833.4), and over half of respondents had deductibles over $2000 and out-of-pocket expenses over $3000, respectively. Lastly, 22.8% of respondents had an HSA and 8.3% had an FSA.

Looking at farm individual and farm household characteristics, 59.5% of respondents reported that at least one household member had a pre-existing or chronic health condition and were on average 57.9 years old. The vast majority (95.7%) of respondents were White, non-Hispanic/Latino, 38.7% were female, 42.9% had at least at bachelor’s degree, 23.3% had children under 18, 47.2% reported an off-farm job within the household, and 7.7% were beginning farmers.

Turning to farm operation characteristics, 39.7% of respondents operated small farms while 21.7% operated medium farms and 38.6% operated large farms. These farms produced grain (53.4%), livestock (37.9%), dairy (20.0%), and fruits and vegetables (13.8%). Over three-quarters of the operations were multi-generational farms.

Finally, when looking at the health and labor environment variables, 56.8% of respondents lived in a state that expanded Medicaid and there were on average 6.8 insurers on the state’s insurance marketplace (standard error 3.3). The unemployment rate in the counties of residence was on average 4.6% (2.0 standard error).

### Prevalence of medical economic vulnerability among surveyed farm households

Starting with the objective measure of economic vulnerability, one in five (20.3%) surveyed farm households had a medical debt of at least $1000 in 2016. The bivariate analysis reveals that few of the independent variables are statistically associated with the debt variable except for the health insurance arrangement variables and two of the individual and household variables (see Table 2 for variables with statistically significant differences; results for all variables are available upon request). Surveyed farm households who reported a medical debt in greater proportion included those who did not have insurance coverage for all members all year (29.1% had a medical debt compared to 19.5% of surveyed households with full coverage), were covered by more than one insurance plan (26.9% compared to 17.9% of households with one plan), had means-based public insurance (31.1% compared to 19.0% of households without means-based public insurance), had higher deductibles (21.9% of households with deductibles over $5000 compared to 14.3% of households with no deductibles), had higher out-of-pocket expenses (28.7% of households with expenses over $5000 compared to 12.2% of households with expenses up to $999), and had a pre-existing or chronic health condition (24.2% compared to 14.6%). Women respondents were also more likely to report that their household had a medical debt (24.0% reported a debt compared to 17.9% of men respondents).

**Table 2:** Farm households with medical debt and who are not confident that they could pay for cost of major illness or injury without going into debt (in % unless otherwise noted)

	Have medical debt (n = 1009)		Not confident (n = 993)	
	Proportion	p	Proportion	p
All farm households	20.3	–	55.4	–
Health insurance arrangements ()				
Coverage for all household members all year		0.048		0.001
Yes	19.5		54.0	
No	29.1		74.1	
All household members had same plan		0.002		n.s.
Yes	17.9		n.s.	
No	26.9		n.s.	
Had medical debt over $1000		n.a.		0.000
Yes	n.a.		66.6	
No	n.a.		52.6	
Off-farm employment		n.s.		0.044
Yes	n.s.		51.4	
No	n.s.		57.9	
Direct purchase of private plan		n.s.		0.042
Yes	n.s.		59.4	
No	n.s.		53.0	
Age-based public health insurance		n.s.		0.000
Yes	n.s.		40.5	
No	n.s.		60.0	
Means-based public health insurance		0.005		n.s.
	31.1		n.s.	
	19.0		n.s.	
Health insurance deductible		0.016		0.000
No deductible	14.3		35.7	
$1 to $1999	15.9		47.8	
$2000 to $5000	25.3		60.2	
More than $5000	21.9		65.8	
Out-of-pocket expenses		0.000		0.001
Up to $999	12.2		47.5	
$1000 to $2999	14.2		49.7	
$3000 to $4999	24.0		65.1	
$5000 and over	28.7		59.3	
Farm individual () and household ()				
Household member(s) with pre-existing or chronic condition	0.000		n.s.	
Yes	24.2		n.s.	
No	14.6		n.s.	
Age (mean)			55.4	0.000
At least one child under 18		n.s.		0.002
Yes	n.s.		63.8	
No	n.s.		52.5	
Female		0.021		0.005
Yes	24.0		61.1	
No	17.9		51.9	
Education		n.s.		0.001
HS or less	n.s.		59.3	
Some college	n.s.		63.5	
Bachelor's degree and higher	n.s.		48.0	
Farm operation ()				
Multi-generational farmer		n.s.		0.002
Yes	n.s.		58.2	
No	n.s.		46.5	
Commodity produced				
Dairy	n.s.	n.s.	63.8	0.007
Fruits and vegetables	n.s.	n.s.	45.7	0.013

*n.a.*. Not applicable, *n.s.*. not statistically significant

Turning to the subjective measure of medical economic vulnerability, over half (55.4%) of surveyed farm households were concerned that they could not pay for the cost of a major illness or injury without going into debt.^8^ The bivariate analysis reveals that more independent variables are statistically associated with the subjective outcome measure than with the objective outcome measure. This includes variables connected to health insurance arrangements. Surveyed farm households who reported not being confident in their ability to pay for major medical expenses in a greater proportion included those who did not have coverage for all members all year (74.1% compared to 54.0%), had a medical debt over $1000 (66.6% compared to 52.6% of households without a debt), did not have insurance coverage through off-farm employment (57.9% compared to 51.4%), had coverage through a private plan (59.4% compared to 53.0%), did not have age-based public health insurance (60.0% compared to 40.5%), had higher deductibles (65.8% of households with deductibles over $5000 compared to 35.7% of households with no deductibles), and had higher out-of-pocket expenses (59.3% of households with expenses over $5000 compared to 47.5% of those with expenses up to $999). Several of the individual, household, and farm operation variables were statistically significant. Farm households more likely to report that they were not confident were on average 55.4 years old (compared to an average age of 60.8 years for those who were confident or neutral), had children under 18 (63.8% were not confident compared to 52.5% for households without children under 18), had lower levels of educational attainment (59.3% for those with a high school degree or less compared to 48.0% for those with a bachelor’s degree or more), were multi-generational farmers (58.2% compared to 46.5% for first generation farmers), were dairy producers (63.8% compared to 53.3% for farm operations that do not produce dairy), and did not grow fruits and vegetables (57.0% compared to 45.7% of farm households grow fruits and vegetables). Last, women respondents were more likely to report not being confident (61.6% compared to 51.9% for men respondents). We note that compared to the objective measures, there is no longer a statistically significant difference between households with pre-existing and chronic conditions compared to those without.

### Factors associated with medical economic vulnerability

Our findings from the logistic regression models indicate that the patterns in statistical significance from the bivariate analysis hold in the multivariate analysis overall (Table 3). Starting with the factors associated with the objective measure of medical economic vulnerability (model 1), only health insurance arrangements and farm individual and household variables were statistically significant. Looking at the health insurance arrangement variables and controlling for the other independent variables, the odds of having a medical debt are 48% lower for households covered by the same insurance plan compared to households with more than one plan. In contrast, the odds of a farm household having a medical debt are 2.21 times higher for those with insurance deductibles between $2000 and $5000 compared to those with none, 74% higher for those with out-of-pocket expenses between $3000 and $4999, and 2.58 times higher for those with out-of-pocket expenses $5000 and over compared to households with expenses up to $999, 49% lower for households with HSA accounts, and 83% higher for those with an FSA account. Looking at individual and farm household variables, the odds of having a medical debt over $1000 are 59% higher for households with pre-existing or chronic conditions compared to those without and increase by 3% for every increase in age, are 63% higher for households with children under 18, and are 47% higher for those with some college compared to those with a high school degree or less.

**Table 3:** Logistic regression predicting the probability of farm households experiencing medical economic vulnerability

	Model 1: objective measure			Model 2: subjective measure			Model 3: subjective measure		
	Have debt over $1000			Not confident that can pay for major health expenses without going into debt			Not confident that can pay for major health expenses without going into debt with holding debt constant		
	Coefficient (standard error)	OR	p	Coefficient (standard error)	OR	p	Coefficient (standard error)	OR	p
Health insurance arrangements ()									
Coverage for all household members all year	− 0.41 (0.39)	0.66	0.289	− **0.93 (0.39)**	**0.39**	**0.017**	− **0.89 (0.38)**	**0.41**	**0.020**
Medical debt over $1000	–	–	–	–	–	–	**0.42 (0.17)**	**1.53**	**0.013**
Source of health insurance (vs. not)									
Off-farm employment	− 0.35 (0.32)	0.70	0.274	− 0.17 (0.20)	0.84	0.383	− 0.15 (0.19)	0.86	0.435
Direct purchase of private plan	− 0.05 (0.22)	0.96	0.834	0.13 (0.21)	1.14	0.539	0.13 (0.21)	1.14	0.533
Age-based public health insurance	− 0.50 (0.31)	0.61	0.104	0.12 (0.23)	1.13	0.600	0.15 (0.23)	1.16	0.512
Means-based public health insurance	0.51 (0.29)	1.67	0.079	0.35 (0.18)	1.42	0.051	0.31 (0.18)	1.36	0.093
Farm Bureau or Farmers' Union	0.11 (0.36)	1.12	0.757	0.27 (0.36)	1.31	0.457	0.26 (0.35)	1.30	0.454
All household has same plan	− **0.66 (0.27)**	**0.52**	**0.014**	**0.41 (0.13)**	**1.50**	**0.002**	**0.45 (0.13)**	**1.57**	**0.000**
Monthly insurance premium	0.00 (0.00)	1.00	0.123	− 0.00 (0.00)	1.00	0.671	− 0.00 (0.00)	1.00	0.621
Health insurance deductible (vs. none)			**0.007**			**0.002**			**0.000**
$1 to $1999	0.12 (0.45)	1.13	0.786	0.43 (0.30)	1.54	0.153	0.41 (0.31)	1.51	0.175
$2000 to $5000	**0.79 (0.45)**	**2.21**	**0.043**	**0.91 (0.29)**	**2.49**	**0.002**	**0.85 (0.29)**	**2.35**	**0.003**
More than $5000	0.53 (0.36)	1.70	0.143	**1.21 (0.38)**	**3.37**	**0.001**	**1.17 (0.38)**	**3.23**	**0.002**
Out-of-pocket expenses (vs. up to $999)			**0.000**			**0.000**			**0.000**
$1000 to $2999	− 0.15 (0.22)	0.86	0.508	− 0.23 (0.15)	0.80	0.133	− 0.21 (0.15)	0.81	0.141
$3000 to $4999	**0.55 (0.17)**	**1.74**	**0.002**	**0.31 (0.15)**	**1.37**	**0.043**	0.29 (0.16)	1.34	0.075
$5000 and over	**0.95 (0.16)**	**2.58**	**0.000**	− 0.07 (0.17)	0.93	0.676	− 0.13 (0.18)	0.88	0.474
Health savings account	− **0.67 (0.20)**	**0.51**	**0.001**	− 0.42 (0.42)	0.65	0.317	− 0.38 (0.41)	0.69	0.358
Flexible spending account	**0.60 (0.21)**	**1.83**	**0.005**	− 0.10 (0.39)	0.91	0.799	− 0.14 (0.40)	0.87	0.732
Individual () and farm household ()									
Pre-existing or chronic condition	**0.46 (0.13)**	**1.59**	**0.000**	**0.29 (0.13)**	**1.34**	**0.025**	**0.27 (0.13)**	**1.31**	**0.033**
Age of respondent	**0.03 (0.01)**	**1.03**	**0.016**	− **0.05 (0.01)**	**0.95**	**0.000**	− **0.05 (0.01)**	**0.95**	**0.000**
Children under 18	**0.49 (0.21)**	**1.63**	**0.029**	− 0.07 (0.20)	0.93	0.713	− 0.10 (0.21)	0.91	0.641
White, non-Hispanic/Latino	0.34 (0.54)	1.40	0.537	− 0.52 (0.28)	0.59	0.058	− **0.54 (0.27)**	**0.58**	**0.041**
Female	0.22 (0.18)	1.25	0.214	0.09 (0.15)	1.10	0.564	0.07 (0.15)	1.01	0.620
Education (vs. high school or less)			**0.002**			**0.028**			0.054
Some college	**0.39 (0.14)**	**1.47**	**0.005**	0.18 (0.14)	1.20	0.187	0.14 (0.14)	1.17	0.271
Bachelor's degree and higher	− 0.00 (0.14)	1.00	0.976	− 0.31 (0.20)	0.74	0.125	− 0.31 (0.20)	0.74	0.127
Off-farm job	0.12 (0.28)	1.13	0.674	0.04 (0.13)	1.04	0.736	0.03 (0.11)	1.03	0.783
Beginning farmer	0.44 (0.34)	1.55	0.199	− **0.45 (0.22)**	**0.64**	**0.040**	− **0.47 (0.22)**	**0.62**	**0.030**
Farm operation ()									
Multi-generational farmer	− 0.16 (0.21)	0.85	0.458	0.32 (0.18)	1.4	0.070	0.33 (0.17)	1.39	0.054
Farm sales (vs. small)			0.115			**0.002**			**0.005**
Medium	− 0.34 (0.29)	0.71	0.233	− 0.26 (0.22)	0.77	0.221	− 0.24 (0.23)	0.78	0.272
Large	− 0.43 (0.25)	0.65	0.084	− **0.43 (0.12)**	**0.65**	**0.001**	− **0.41 (0.13)**	**0.66**	**0.002**
Commodity produced (vs. not)									
Grain	− 0.21 (0.26)	0.81	0.422	0.15 (0.20)	1.16	0.453	0.17 (0.20)	1.19	0.414
Livestock	− 0.16 (0.25)	0.85	0.524	0.16 (0.25)	1.17	0.531	0.17 (0.25)	1.18	0.484
Dairy	0.42 (0.48)	1.52	0.383	**0.56 (0.19)**	**1.74**	**0.004**	**0.54 (0.19)**	**1.72**	**0.004**
Fruits and vegetables	0.43 (0.33)	1.54	0.191	− 0.02 (0.18)	0.98	0.919	− 0.04 (0.18)	0.96	0.835
Health insurance and labor market () environments									
State expanded Medicaid	− 0.46 (0.34)	0.63	0.175	− 0.33 (0.22)	0.72	0.145	− 0.30 (0.21)	0.74	0.150
Number insurers on marketplace	0.01 (0.04)	1.01	0.877	0.04 (0.02)	1.04	0.080	0.04 (0.02)	1.04	0.079
Unemployment rate	0.05 (0.05)	1.05	0.284	− 0.01 (0.03)	1.00	0.802	− 0.01 (0.03)	0.99	0.717
Constant	− 3.47 (1.18)	0.03	0.003	3.07 (0.92)	21.52	0.001	3.08 (0.91)	21.78	0.001
Model F-test	F (36, 5574.9) = 48.81			F (35, 4445.5) = 97.80			F (36, 4434.1) = 90.43		
Model p-value	0.000			0.000			0.000		
Adjusted R^2^	7.7			11.3			11.7		

Examining the factors associated with the subjective measure (models 2 and 3 where debt is added as an independent variable in model 3), the significance of health insurance arrangement variables and individual and farm household characteristics shifted while variables connected to the farm operation became statistically significant. As discussed in the methods section, the addition of the debt variable improved model fit, and therefore, we focus our interpretation of the findings on model 3. Controlling for the other independent variables, the odds of not being confident in the ability to pay for the cost of a major illness or injury without going into debt are 59% lower for households with health insurance coverage for all members all year. Conversely, the odds of not being confident in having the ability to pay for a major illness or injury are 53% higher for households with a medical debt over $1000, 57% higher for households covered under the same plan, 2.35 times higher for those with deductibles between $2000 and $5000, and 3.23 times higher for those with deductibles $5000 and over compared to households with no deductibles. Turning to farm household demographics, the odds of not being confident in the ability to pay for major health expenses are 31% higher for households with a pre-existing condition, decrease by 5% for every increase in age, are 42% lower for White, non-Hispanic/Latino respondents, and are 38% lower for beginning farmers. Looking at farm operation variables, the odds of not being confident in the ability to pay are 34% lower for large-scale farm operations compared to small farms and 72% higher for dairy operations compared to non-dairy farming operations. Compared to model 1 with the objective outcome measure, variables connected to out-of-pocket expenses, having HSA and FSA accounts, having children under 18, and having a college education are no longer statistically significant.

## Discussion

Our three research questions aimed to assess the prevalence and factors associated with medical economic vulnerability among a sample of over 900 U.S. farm families. We now synthesize our results by comparing and contrasting our results with the literature, including the literature that formed the basis of our conceptual framework. We consider the larger implications of our results within the context of the broader farm resilience literature and current limitations in the conclusion.

### Prevalence of medical economic vulnerability and variations based on measures used

Our first research question aimed to assess the prevalence of U.S. farm households experiencing medical vulnerability. We used two measures (one objective, the other subjective) of vulnerability in response to farm resilience scholars’ calls to move beyond the current focus on objective measures by analyzing subjective measures that speak to farmers’ realities and perceptions (Darnhofer et al. 2016; Meuwissen et al. 2019; Perrin et al. 2020). Our univariate analysis points to important differences in the prevalence of medical economic vulnerability for the two measures. Though only one in five surveyed farm households had a medical debt over $1000 in 2016, more than half were not confident that they could pay for a major health expense without going into debt. In other words, the concern of having to take on a debt in anticipation of a major medical problem was 2.7 times higher than having a medical debt over $1000. The discrepancy between objective and subjective outcome measures have been found both in the medical economic vulnerability among the general population (Asebedo and Wilmarth 2017; Banegas et al. 2016) and in surveys of farmers pre-ACA (Dulitz and Schrader 2013; Pryor et al. 2008, 2009). The finding of a general sense of medical economic vulnerability based on the subjective measure also connects back to two bodies of literature. First, the health shock literature from low and medium-income countries has found high prevalence of medical vulnerability that are in some cases higher than farm-operation level challenges such as weather events or pest pressure (Alam and Mahal 2014; Bonfrer and Gustafsson-Wright 2017). This body of literature is important since besides a few studies pre-ACA in the U.S., we are not aware of studies on medical economic vulnerability among the agricultural population in high-income countries. Second, the U.S. farm stress literature has found that health-related expenses are a stressor in magnitude similar to farm-related stressors such as cost of land and farm input (Fraser et al. 2005; Inwood 2015; Jackson-Smith et al. 2021).

### Limited variations in who experiences medical economic vulnerability

Our second research question aimed at assessing who is more likely to experience medical economic vulnerability on the basis of farm individual and household demographics as well as farm characteristics (i.e. the role of micro-level spheres within the agri-family system). Despite a somewhat heterogeneous sample of farm families, our multivariate analysis indicates that few variables are statistically significantly associated with the two measures of economic vulnerability with some variations across the objective and subjective measures. In line with previous research (Baughman et al. 2015; Hamel et al. 2016; Katchova and Dinterman 2018; Pryor et al. 2008, 2009; Wiltshire et al. 2016), having a pre-existing or chronic health condition was associated with both outcome measures. In contrast, having children under 18 and lower educational attainment was associated with having a medical debt while White, non-Hispanic respondents, farmers not operating a dairy farm, and operating a large operation (compared to a small operation) were associated with being confident, or neutral, in their ability to pay for major expenses without going into debt. The increase in having a medical debt as respondents age coupled with the decrease in not being concerned in ability to pay for major expenses is similar to the mixed findings around age in previous research (Baughman et al. 2015; Hamel et al. 2016; Himmelstein et al. 2009; Pryor et al. 2008, 2009) and highlights differences based on outcome measures. The difference in the sign of the association may be explained by the increase in health care needs and accumulation of debt over the years while farmers eligible for age-based public insurance have previously reported lower level of challenges paying for health expenses (Dulitz and Schrader 2013; Lottero et al. 2007). Lastly, a greater level of confidence among beginning farmer respondents to pay for medical expenses differs from the farm economic stress literature (Katchova and Dinterman 2018). Our finding may be explained by the lower age of beginning farmer respondents and/or a healthy worker effect (i.e., those self-selecting to enter agriculture are more likely to be healthy). Indeed, one-third of beginning farmer respondents reported a pre-existing or chronic health condition compared to two-thirds of non-beginning farmer respondents.

The overall limited variation in who experiences medical economic vulnerability on the basis of demographic and farm characteristics is a counterpoint to farm resilience assessments that at times place a heavy emphasis on these individual-level variables. Instead, our results align with Chang et al.’s (2011) finding that demographic characteristics played a limited role in health-related early exit from agriculture. This finding could in part be reflective of the dangerous and physical nature of agricultural work, which is captured in our dataset (e.g. approximately 60% of respondents reported at least one household member with a pre-existing or chronic health condition). The limited variation may also be explained by the general sense of medical economic vulnerability found both in our sample and pre-ACA farmer surveys (Dulitz and Schrader 2013; Pryor et al. 2008, 2009). Meanwhile, we note that three of the demographic and farm characteristics that are statistically significant for the subjective measure (i.e. beginning farmer status, farm sales, and dairy production) can be acted on through federal-level agricultural programs in the Farm Bill. Though we did not ask about participation in farm programs, our findings reinforce the importance of assessing systems’ features that support farm resilience (Darnhofer 2021) including the need for future research aimed at understanding the role that agricultural policy may play in shaping medical economic vulnerability.

### Importance of health insurance arrangements in shaping medical economic vulnerability

Our third research question aimed to assess the role of institutional arrangements in medical economic vulnerability with a focus on health insurance coverage (i.e. role of macro-level factors). Our univariate analysis points to the on-going problem of underinsurance (Pryor et al. 2008, 2009). Indeed, despite over 9 in 10 respondents having health insurance coverage for all household members all year, 1 in 5 reported a medical debt and over half are not confident in their ability to pay for major medical expenses. Second, our multivariate analysis hints at the importance of health insurance relative to demographic and farm operation characteristics as seen through a comparison of the size of the statistically significant coefficients. Out of the five largest coefficients for the two outcome measures, four of these coefficients were for health insurance variables. This finding parallels studies examining medical economic vulnerability among the general population (Hamel et al. 2016; Himmelstein et al. 2005).

Our multivariate analysis also indicates that experiencing medical economic vulnerability is not simply a matter of having health insurance coverage but also a matter of the type of coverage a household has with variations based on outcome measures. For example, reporting that all households were covered on the same plan decreased the probability of having a medical debt but increased the probability of not being confident in the ability to pay for major expenses. Another example comes from variables connected to health-related expenses. The cost of a health insurance premium was not statistically significant for either outcome variables though the level of financial coverage (i.e., deductibles, out-of-pocket expenses, and HSAs) varied across the two models.

Overall, there are important discrepancies between our findings and previous studies related to the relationships between medical economic vulnerability and health insurance coverage, source of coverage, and health insurance environment (Banegas et al. 2016; Baughman et al. 2015; Frank and McGuire 2017; Mazurenko et al. 2018; Pryor et al. 2009). There are several potential explanations for these discrepancies. First, in pre-ACA studies of farmers, health insurance arrangement variables were limited to health insurance coverage, source, and health expenses. The association between medical debt and health insurance coverage might not be present in our study due to the greater level of granularity in the measures we used. This highlights the importance of including variables that speak to the specifics of health insurance coverage in future studies to fully assess the role of health insurance arrangements. The lack of significance in our study for the macro-level health insurance variables could be in part connected to the small number of study states. It could also be indicative of the relatively limited variations across states in health insurance environments since federal regulations limit the range of variations in terms of what private health insurers and states can offer. The discrepancy may also be explained by variations in outcome variables used across studies, especially for the subjective measure.

## Conclusion

In response to contemporary crises, including climate change, trade liberalization, and more recently the COVID-19 pandemic, the farm resilience literature has expanded greatly in recent years with the central objective of understanding farm families’ ability to continue farming despite on-going challenges. Yet, two major limitations of this literature, namely, a focus on macro-level challenges faced by the farm operation and a mismatch between the scale of challenges studied (i.e. macro-level) and resilience measures (i.e. largely focused on micro-level variables), likely limit our understanding of the range and frequency of challenges that farm families face and the range of factors that shape resilience. These limitations then call into question our ideas about the types of interventions needed to support the farm sector and farm families’ ability to survive a crisis.

In this study, we expand the range of challenges examined in the farm resilience literature by using the example of medical economic vulnerability, a micro-level challenge traditionally confined to the household sphere of the agri-family system. Our conceptual framework links the medical economic vulnerability and farm economic stress bodies of literature with the multi-scalar agri-family system as the backdrop to provide a space to examine the interplay between individual-level factors and features of the bigger system in which farms are embedded. Through an analysis of over 900 surveys of farm households from 10 U.S. states, we found seemingly large differences in the prevalence of medical economic vulnerability across the objective and subjective outcome measures, with the subjective measure indicating a sentiment of medical economic vulnerability among the majority of surveyed respondents. Conversely, there was limited variation in who experiences medical vulnerability based on demographic and farm characteristics while the strongest associations are connected to a households’ health insurance arrangements.

Our findings have three main implications for the farm resilience literature. First the presence of medical economic vulnerability among a significant proportion of surveyed farm families (especially for the subjective measure) reinforces recent calls to expand the conceptualization of what constitutes challenges in the resilience literature (Komarek et al. 2020; Popp and Nowack 2020). Our findings point to the importance of including micro-level challenges that impact the farm household. Our study was focused on assessing the prevalence and factors associated with medical economic vulnerability, the next step is to understand how medical economic vulnerability interacts with and affects farm resilience in the medium- and long-term. Understanding farm resilience and building more resilient food and agriculture systems also requires us to grapple with how other micro-level challenges (e.g. a barn fire, loss of off-farm employment, or divorce) impact the farm operation or the household and affect farm resilience.

Second, the variation in results between objective and subjective outcome measures reinforces the importance of using multi-dimensional measurements attentive to both quantitative thresholds deemed indicative of challenges, as well as farmers’ lived realities and their perception of current and future prospects (Darnhofer et al. 2016; Meuwissen et al. 2019; Perrin et al. 2020; Rissing 2019). Based on our findings, discounting farm households’ lived experience could lead to situations where we overlook the early warning signs of a looming crisis. It could also lead to situation where we ignore low-grade challenges, even if they do not meet the objective measure threshold, that may still erode resilience in the long-term. Inherent in this work is the need to better understand how researchers and practitioners can utilize paired objective and subjective measures to understand current and emerging issues, and the broader types of investments and interventions that contribute to farm resilience at different scales. Since farm resilience studies incorporating subjective measures have largely been qualitative (Daugstad 2019; Perrin et al. 2020), our study provides an example of how objective and subjective measures can be combined in the same quantitative study.

Third, the significant role health insurance arrangements and potential role agricultural policies play in shaping medical economic vulnerability underscore the importance of considering factors beyond the micro-level factors. These findings reinforce critiques of the resilience literature and bolster the need for researchers to account for a broader range of factors that individual farmers do not directly control including meso- and macro-level factors (Calo 2020; Cote and Nightingale 2012; Joseph 2013). Our empirical case is situated in a country with strong farm income support for some sub-agricultural populations compared to some countries (e.g. Australia, Chile, and New Zealand) but with a limited social safety net compared to most other Western industrialized nations. Yet, research from Europe, Canada, and Australia indicates that even with stronger social policies, farm households experience challenges meeting their social and economic needs (Chappuis et al. 2015; Contzen and Crettaz 2019; Contzen et al. 2016; Courtenay Botterill 2007; Droz et al. 2014; Roche 2016). Collectively, these findings reinforce contemporary calls (Becot and Inwood 2020; Darnhofer 2021) to expand the types of issues and variables farm family scholars study. This includes the need to research interactions between institutional arrangements connected to social and agricultural policies and farm resilience.

## Abbreviations

ACA: Patient Protection and Affordable Care Act
ARMS: Agricultural Resource Management Survey
COVID-19: Coronavirus Disease 2019
EFA: Exploratory factor analysis
FSA: Flexible spending accounts
HSA: Health savings account
MICE: Multiple imputation by chained equation
U.S.: United States
USDA: United States Department of Agriculture
VIF: Variance inflation factor

## Footnotes

1 For a review of how the ACA re-shaped the health insurance landscape and how it was expected to impact the farm sector see Ahearn et al. (2015) and Inwood (2017).

2 At the time of our study in the early years of implementation of the ACA, a federal mandate required all individuals to either have health insurance or pay a tax penalty. This mandate ended in 2019 but five states have since then instituted a state-level mandate. Farmers can obtain health insurance from a range of sources including from the federal government for aged-based universal coverage starting at 65 or for individuals with a disability, state-level government for means-based coverage, through their off-farm employer, and through the purchase of a plan from a private insurer. Readers not familiar with the U.S. health insurance system can refer to Field (2017) and Rice et al. (2013) for a general overview.

3 The three Likert-scale variables used in the EFA are as follows: (1) Health insurance meets needs, (2) Confidence that can afford the usual medical costs they currently have (assuming no emergency), (3) Confidence that can pay medical costs, without going into debt, if had a major illness or injury.

4 HSA and FSA accounts are savings accounts that provide tax advantages for eligible health expenses. Only those with a high deductible health plan are eligible for an HSA account while only those with an insurance group or employer plan are eligible for an FSA account.

5 USDA categories based on farm sales: hobby: less than $10,000 (category was removed from our sample), small: between $10,000 and $249,999, medium: between $250,000 and $499,999, and large: $500,000 and over.

6 To create a household income estimate to assess potential specification errors in the multivariate analysis, Daniel Prager, previously at the USDA, extracted farmer responses located in the 10 states from the 2016 ARMS dataset (except for 2 states where regional data were used). He then estimated a range of household income estimates for this sub-sample using a combination of five variables collected both in the ARMS survey and ours: (1) off-farm employment, (2) total farm sales ≥ $250 K, (3) educational attainment of at least some college, (4) primary operator is ≥ 65 years of age, (5) beginning farmer status). We then merged this newly estimated household income in our dataset by matching the five variable combination.

7 Multilevel analysis can be indicated to parse out the effects of variables at various levels of analysis. We did not have the generally recommended number of clusters (at least 20) to conduct this type of analysis (Hox 2010).

8 We collapsed this variable into a dummy variable for the bivariate and multivariate analysis but 31.7% of respondents were moderately or very confident that they could pay for a major illness or injury without going into debt and 12.9% were neutral.
